# Conversational AI in Pediatric Mental Health: A Narrative Review

**DOI:** 10.3390/children12030359

**Published:** 2025-03-14

**Authors:** Masab Mansoor, Ali Hamide, Tyler Tran

**Affiliations:** Edward Via College of Osteopathic Medicine—Louisiana Campus, Monroe, LA 71203, USA; ahamide@ulm.vcom.edu (A.H.); ttran02@ulm.vcom.edu (T.T.)

**Keywords:** conversational AI, chatbots, large language models, mental health, pediatrics

## Abstract

Background/Objectives: Mental health disorders among children and adolescents represent a significant global health challenge, with approximately 50% of conditions emerging before age 14. Despite substantial investment in services, persistent barriers such as provider shortages, stigma, and accessibility issues continue to limit effective care delivery. This narrative review examines the emerging application of conversational artificial intelligence (AI) in pediatric mental health contexts, mapping the current evidence base, identifying therapeutic mechanisms, and exploring unique developmental considerations required for implementation. Methods: We searched multiple electronic databases (PubMed/MEDLINE, PsycINFO, ACM Digital Library, IEEE Xplore, and Scopus) for literature published between January 2010 and February 2025 that addressed conversational AI applications relevant to pediatric mental health. We employed a narrative synthesis approach with thematic analysis to organize findings across technological approaches, therapeutic applications, developmental considerations, implementation contexts, and ethical frameworks. Results: The review identified promising applications for conversational AI in pediatric mental health, particularly for common conditions like anxiety and depression, psychoeducation, skills practice, and bridging to traditional care. However, most robust empirical research has focused on adult populations, with pediatric applications only beginning to receive dedicated investigation. Key therapeutic mechanisms identified include reduced barriers to self-disclosure, cognitive change, emotional validation, and behavioral activation. Developmental considerations emerged as fundamental challenges, necessitating age-appropriate adaptations across cognitive, emotional, linguistic, and ethical dimensions rather than simple modifications of adult-oriented systems. Conclusions: Conversational AI has potential to address significant unmet needs in pediatric mental health as a complement to, rather than replacement for, human-delivered care. Future research should prioritize developmental validation, longitudinal outcomes, implementation science, safety monitoring, and equity-focused design. Interdisciplinary collaboration involving children and families is essential to ensure these technologies effectively address the unique mental health needs of young people while mitigating potential risks.

## 1. Introduction

Mental health disorders represent one of the most significant health challenges facing children and adolescents globally. Despite substantial investments by governments and healthcare systems over the past two decades, the prevalence of mental health conditions among young people has remained stubbornly unchanged [1]. This fact is particularly concerning given that approximately 50% of all mental health disorders have their onset before the age of 14 years [2]. The COVID-19 pandemic has further exacerbated this situation, introducing new stressors and barriers to care while simultaneously accelerating digital transformations in healthcare delivery [3,4].

Traditional models of pediatric mental healthcare continue to face persistent challenges. Long waiting lists, high costs, geographical limitations, stigma, and shortages of specialized providers create substantial barriers to accessing timely and appropriate care [5]. These systemic issues are further complicated by difficulties in early identification of mental health concerns in children and adolescents, as well as the tendency for pediatric mental health services to mirror adult-oriented approaches that may not adequately address the unique developmental needs of younger populations [6,7].

Against this backdrop, artificial intelligence (AI) conversational agents—such as large language models (LLMs) exemplified by ChatGPT, Claude, and similar technologies—have emerged as potential supplementary tools in the mental health landscape [8,9]. These AI systems can engage in text-based dialogues that simulate human conversation, providing a novel medium through which children and adolescents might express emotions, receive information, and potentially experience therapeutic interactions [10]. The 24/7 availability, non-judgmental interaction environment, and low access threshold of these tools address several traditional barriers to care, suggesting potential utility as adjunctive approaches within comprehensive mental health frameworks [11].

However, the application of conversational AI in pediatric mental health contexts remains in its infancy, with limited empirical investigation specifically addressing efficacy, safety, and implementation considerations for children and adolescents [12,13]. While preliminary research with adult populations has indicated promising results for certain applications—such as cognitive behavioral therapy support, mood monitoring, and psychoeducation [14]—the translation of these findings to pediatric populations requires careful consideration of developmental, ethical, and safety factors [15].

This review aims to critically examine the current evidence base regarding conversational AI applications in pediatric mental health, synthesizing findings across disciplines including psychology, psychiatry, computer science, and ethics. We evaluate the potential therapeutic mechanisms through which these tools might function, identify promising applications within pediatric mental health contexts, and discuss critical considerations for safe and effective implementation. By mapping the current state of knowledge and identifying key research gaps, this review seeks to establish a foundation for future empirical work while providing preliminary guidance for clinicians, developers, and policymakers interested in the responsible integration of AI conversational agents into pediatric mental health services [16].

### Research Questions

This narrative review aimed to address the following questions:

1. What types of conversational AI applications have been developed or proposed for supporting pediatric mental health?
2. What is the current evidence regarding the effectiveness, acceptability, and safety of these applications?
3. What unique considerations apply to the use of conversational AI with children and adolescents compared to adults?
4. What ethical, technical, and implementation challenges have been identified?
5. What gaps exist in the current research landscape?

## 2. Methods

### 2.1. Information Sources and Search Strategy

We conducted searches across multiple electronic databases to capture literature from diverse disciplines, including medicine, psychology, computer science, and digital health. Databases included PubMed/MEDLINE, PsycINFO, ACM Digital Library, IEEE Xplore, and Scopus. The search was supplemented by examining reference lists of included articles and relevant review papers.

The search strategy combined terms related to three key concepts: (1) conversational AI technologies (e.g., “chatbot”, “conversational agent”, “large language model”), (2) mental health (e.g., “mental health”, “psychological support”, “therapy”), and (3) pediatric populations (e.g., “child”, “adolescent”, “youth”). The search was limited to English-language publications from January 2010 to February 2025. Given the rapid evolution of large language models, we also included relevant preprints from arXiv and other repositories to capture emerging research.

### 2.2. Study Selection

Articles were eligible for inclusion if they discussed conversational AI applications in mental health contexts, had relevance to pediatric populations (either directly studying children/adolescents or with clear pediatric implications), and included original research, theoretical frameworks, ethical analyses, or significant commentary on implementation. We included a broad range of publication types, including empirical studies, technical descriptions, ethical analyses, theoretical papers, and relevant case studies. Initial screening of titles and abstracts was conducted by two independent reviewers, with full-text review of potentially relevant articles. Our initial database search yielded 842 potentially relevant articles. After removing duplicates and screening titles and abstracts, 156 articles underwent full-text review, with 87 meeting final inclusion criteria for analysis.

### 2.3. Data Synthesis

We employed a narrative synthesis approach with thematic analysis to organize findings into meaningful categories. The analysis focused on mapping the current landscape rather than evaluating effectiveness or quality. We identified patterns across studies while highlighting conceptual boundaries, contradictions in the literature, and areas of emerging consensus. Special attention was given to identifying research gaps and future directions.

The synthesis was structured thematically around key aspects of conversational AI in pediatric mental health, including technological approaches, therapeutic applications, developmental considerations, implementation contexts, and ethical frameworks. This organization allows for a comprehensive overview of the field accessible to interdisciplinary audiences.

## 3. Current Landscape of Pediatric Mental Health Challenges

Mental health disorders represent a significant and growing public health concern for children and adolescents worldwide. Current epidemiological data indicate that approximately 13–20% of children under 18 years of age experience a diagnosable mental health condition in any given year [17]. More concerning is evidence suggesting that the prevalence of certain conditions, particularly anxiety, depression, and behavioral disorders, has been steadily increasing over the past decade [18]. This trend has been further accelerated by the COVID-19 pandemic, which introduced unprecedented stressors including social isolation, academic disruptions, family economic hardships, and grief experiences [19].

The burden of pediatric mental health conditions is distributed unevenly across populations, with significant disparities observed across socioeconomic, racial, and geographic lines [20]. Children with multiple marginalized identities often face compounded challenges, as the intersection of disability status, gender identity, sexual orientation, immigration status, and language barriers can create unique barriers to accessing appropriate care. Children from lower-income families, racial and ethnic minorities, and those living in rural or underserved communities face disproportionate challenges in both the prevalence of mental health conditions and access to appropriate care [21]. These disparities reflect broader social determinants of health that influence both the development of mental health conditions and the barriers to receiving timely intervention [22]. Despite clear evidence demonstrating the benefits of early intervention, substantial gaps persist between the need for mental health services and their availability and accessibility for children and adolescents [23].

### Challenges of Current Mental Health Delivery

Current systems of care struggle with multiple interconnected challenges that limit their effectiveness.

- Workforce Shortages: The global shortage of child psychiatrists, psychologists, and specialized mental health professionals creates a fundamental capacity limitation [24]. In many regions, the ratio of child mental health specialists to the pediatric population falls dramatically below recommended levels, creating bottlenecks in service delivery and extending wait times for initial assessments and ongoing treatment [25].
- Access Barriers: Even when services exist, multiple barriers impede access, including geographical limitations, transportation challenges, scheduling constraints, high costs, and insurance coverage limitations [26]. For many families, particularly those in rural or underserved areas, the nearest appropriate provider may be hours away, making regular attendance at appointments impractical or impossible [27].
- Fragmented Systems: Pediatric mental healthcare often spans multiple systems including healthcare, education, juvenile justice, and social services. Poor coordination between these systems creates fragmented care pathways, administrative burdens for families, and opportunities for vulnerable children to “fall through the cracks” [28].
- Detection and Referral Challenges: Many mental health conditions in children present initially as somatic complaints, behavioral problems, or academic difficulties and may not be readily recognized as mental health concerns by parents, teachers, or primary care providers [29]. These challenges contribute to delays in identification and appropriate referral, particularly in contexts where mental health literacy remains limited [30].
- Stigma and Help-Seeking Barriers: Despite some progress, mental health conditions continue to carry significant stigma that can discourage help-seeking behaviors among young people and their families [31]. Adolescents, in particular, often express concerns about confidentiality, judgment from peers, and reluctance to engage with traditional clinical environments [32].
- Developmental Considerations: Children’s mental health needs vary significantly across developmental stages, requiring age-appropriate assessment tools and intervention approaches that many systems struggle to properly differentiate and implement [33]. What works for an adolescent may be entirely inappropriate for a young child, yet services often lack the flexibility to adequately address these differences [34].
- Treatment Adherence and Engagement: Even when children do access care, engagement and adherence challenges are common, with dropout rates from traditional mental health services estimated at 40–60% [35,36]. Factors contributing to poor engagement include practical barriers, perceived lack of cultural competence, misalignment with youth preferences, and failure to involve families effectively [37].

The pandemic has both exacerbated these existing challenges and catalyzed innovation in service delivery models. Telehealth adoption has accelerated dramatically, demonstrating that remote care options can effectively reach some previously underserved populations [38]. However, the rapid shift to digital platforms has also highlighted the “digital divide”, with families lacking adequate technology or internet access experiencing new barriers to care [39].

Despite growing identification of evidence-based treatments for various pediatric mental health conditions, implementation lags significantly, particularly for complex presentations or co-occurring disorders. The availability of specialists trained in evidence-based approaches for conditions such as trauma, OCD, eating disorders, or autism with co-occurring psychiatric conditions remains especially limited.

These complex and interrelated challenges in pediatric mental healthcare create both an urgent need and a fertile environment for innovative approaches that can complement traditional services, address specific access barriers, and potentially reach children and families who remain underserved by current systems [40]. It is within this context that conversational AI applications have emerged as a potential component of more accessible, scalable, and youth-friendly mental health support infrastructure [41].

## 4. Emergence of AI Conversational Agents in Mental Health

The integration of artificial intelligence into mental healthcare represents a significant evolution in digital mental health interventions. While digital tools for mental health have existed for decades—from computerized cognitive behavioral therapy programs to mobile applications for mood tracking—AI conversational agents mark a qualitative shift in how technology can simulate human-like therapeutic interactions [42].

### 4.1. Historical Context and Technological Evolution

The conceptual foundation for conversational agents in mental health can be traced back to ELIZA, a computer program developed by Joseph Weizenbaum at MIT in the 1960s [43]. ELIZA simulated a Rogerian psychotherapist using simple pattern-matching techniques to reflect user statements back as questions, creating an illusion of understanding. Despite its technical limitations, ELIZA demonstrated the potential for computer programs to engage users in therapeutic-like conversations and revealed people’s tendency to anthropomorphize and disclose personal information to computer systems [44].

Early rule-based chatbots for mental health that followed ELIZA relied on predefined scripts and decision trees, offering limited flexibility in conversations [45]. The next generation incorporated more sophisticated natural language processing capabilities but still operated within relatively constrained conversational parameters [46]. These systems showed promise in specific applications such as screening for mental health conditions, delivering psychoeducational content, and guiding users through structured therapeutic exercises [47].

**Figure 1:**
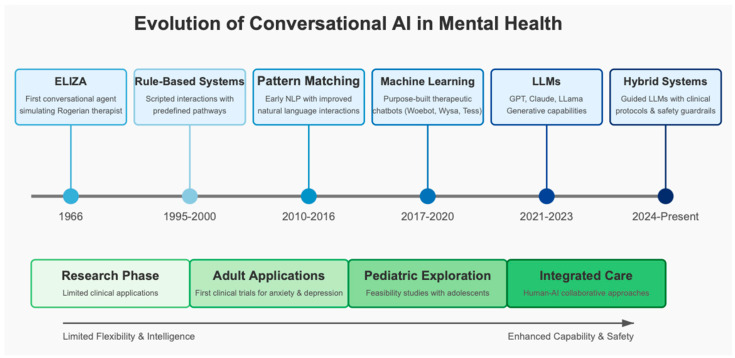
Evolution of conversational AI technologies in mental health (1966–present). This timeline illustrates the progression from early rule-based systems like ELIZA to modern hybrid approaches combining large language models with clinical protocols. The lower section shows the parallel evolution of therapeutic applications, from the initial research phase through adult applications to pediatric exploration and current integrated care approaches. This technological trajectory has been characterized by increasing capability, flexibility, and safety features, particularly important for pediatric mental health applications.

The landscape transformed dramatically with the advent of large language models (LLMs) based on transformer architectures, exemplified by systems like GPT (Generative Pre-trained Transformer), Claude, and similar technologies [48]. These models, trained on vast corpora of text from the internet and other sources, can generate contextually relevant, coherent responses without explicit programming for specific scenarios [49]. This technological leap enabled conversational agents to engage in more natural, flexible dialogues across a wide range of topics, including sensitive mental health conversations [50]. As illustrated in Figure 1, conversational AI technologies have evolved significantly since ELIZA in the 1960s, with recent developments in large language models and hybrid systems offering unprecedented capabilities while raising new considerations for pediatric applications.

### 4.2. Types of Conversational AI in Mental Health Applications

Current conversational AI systems in mental health contexts can be categorized based on their technological sophistication, therapeutic approach, and intended function, as visible in Table 1.

- Rule-Based Systems: Rule-based systems follow predetermined conversation flows and decision trees [51]. While limited in handling unexpected user inputs, they offer precise control over therapeutic content and safety guardrails [52]. Examples include Woebot and Wysa, which deliver structured cognitive behavioral therapy exercises through guided conversations [53].
- Retrieval-Based Systems: Retrieval-based systems select appropriate responses from a database based on user input patterns. They provide more flexibility than rule-based systems while maintaining consistency in therapeutic approaches [54].
- Generative AI Systems: Generative AI systems generate novel responses based on patterns learned during training rather than selecting from predefined options [55]. Modern LLMs fall into this category, offering unprecedented conversational flexibility but raising questions about consistency and safety [56].
- Hybrid Approaches: Many deployed systems combine elements of these approaches, using rule-based frameworks to guide the overall therapeutic structure while employing generative or retrieval-based techniques for specific conversational components [57].

**Table 1:** Comparison of conversational AI system types used in mental health applications, including their key characteristics, examples, strengths, limitations, and special considerations for pediatric applications.

Type	Key Characteristics	Examples in Mental Health	Strengths	Limitations	Pediatric Considerations
Rule-Based Systems	Predetermined conversation flows and decision trees; Script-based interactions	Woebot, Wysa	Precise control over therapeutic content; Safety guardrails; Consistent delivery of interventions	Limited flexibility with unexpected user inputs; May feel mechanical; Cannot easily adapt to novel situations	Can be designed with age-appropriate content; Safety-focused; Less likely to generate inappropriate responses
Retrieval-Based Systems	Select responses from existing database based on user input patterns	Tess, Replika (in more structured modes)	More flexible than rule-based systems; Consistency in therapeutic approach; Can incorporate evidence-based responses	Limited to existing response database; Less adaptable to unique user needs	Can incorporate developmentally appropriate response sets; May struggle with child-specific language patterns
Generative AI Systems	Generate novel responses based on training data patterns; Not limited to predefined responses	ChatGPT, Claude when used for mental health support	Highly flexible conversations; Can address unexpected inputs; More natural dialogue flow	Less predictable responses; Safety and accuracy concerns; Potential for harmful content	Higher risk of developmentally inappropriate responses; Require substantial safety measures; Often trained primarily on adult language
Hybrid Approaches	Combine rule-based frameworks with generative or retrieval capabilities	Emerging integrated platforms; Wysa’s newer versions	Balance of structure and flexibility; Combine safety controls with conversational naturalness	More complex to develop and maintain; May have inconsistent interaction quality	Potentially optimal for pediatric applications; Can incorporate developmental safeguards while maintaining engagement

The technological approaches described above enable different functional roles in mental health contexts, with rule-based systems often excelling at structured therapeutic protocols, while generative systems may better facilitate open-ended emotional support conversations. 

Functionally, these systems serve various roles in mental health contexts:

- **Screening and Assessment Tools**: Conversational interfaces that gather information about symptoms and experiences to support early identification of mental health concerns [58].
- **Psychoeducational Agents**: Systems that provide information about mental health conditions, coping strategies, and available resources through interactive dialogue rather than static content [59].
- **Guided Self-Help Programs**: Structured therapeutic interventions delivered through conversational interfaces, often based on evidence-based approaches like cognitive behavioral therapy [60].
- **Emotional Support Companions**: Applications designed primarily for empathetic listening and validation rather than formal therapeutic interventions [61].
- **Adjuncts to Traditional Therapy**: Tools that complement professional care by supporting homework completion, skills practice, or monitoring between sessions [62].

### 4.3. Initial Evidence in Adult Populations

Research on conversational AI for mental health has predominantly focused on adult populations, with pediatric applications emerging more recently. Several review studies and meta-analyses have established a foundational understanding of these technologies’ potential in mental health contexts.

The evidence framework for conversational AI in mental health has evolved from case studies and feasibility assessments to more rigorous controlled trials, primarily with adult users. These studies have collectively established proof-of-concept for several potential therapeutic mechanisms, including accessibility advantages, enhanced disclosure, and consistent delivery of therapeutic techniques. Additionally, researchers have identified important implementation factors, such as user engagement patterns, privacy considerations, and integration with traditional care pathways.

Investigations of user experiences have revealed that the perception of non-judgment and anonymity appears to facilitate self-disclosure, particularly regarding stigmatized topics. The 24/7 availability addresses practical barriers to traditional care, while the conversational format may feel more natural and engaging than other digital interventions.

These foundational findings with adult populations have established sufficient proof-of-concept to justify exploration in pediatric contexts, while simultaneously highlighting the need for age-specific adaptations and careful consideration of developmental factors in implementation. The transition from adult-focused to pediatric applications represents not merely a change in target population but necessitates fundamental reconsideration of content, interaction patterns, safety protocols, and ethical frameworks.

The following section examines the specific evidence for efficacy in both adult and pediatric populations in greater detail, with particular attention to methodological approaches and outcome measures relevant to each developmental stage.

## 5. Evidence for Efficacy and Therapeutic Mechanisms

The evidence base for conversational AI applications in mental health is still developing, with significant variations in methodological rigor, study design, and outcome measures across investigations. This section examines the current state of evidence regarding both efficacy and the potential therapeutic mechanisms through which these systems may influence mental health outcomes.

### 5.1. Evidence in Adult Populations

The most robust empirical research on conversational AI for mental health has been conducted with adult populations, providing important methodological frameworks and baseline effectiveness data that inform pediatric applications. Across multiple randomized controlled trials, several patterns have emerged.

Common mental health conditions, including depression and anxiety, have received the most research attention, with multiple studies demonstrating small to moderate positive effects compared to waitlist or information-only controls. For example, Fitzpatrick et al. [60] conducted an RCT of Woebot, a CBT-based chatbot, with 70 young adults experiencing symptoms of depression and anxiety. After two weeks, participants using Woebot showed significantly greater reductions in depression symptoms compared to an information control group (effect size d = 0.44). Engagement metrics were promising, with participants having an average of 12 conversations with the bot over the study period. In a larger trial, Fulmer et al. [63] evaluated a conversational agent delivering behavioral activation techniques to 75 adults with mild to moderate depression symptoms. The intervention group showed significantly greater improvement in depression scores at 4-week follow-up compared to a waitlist control, with improvements maintained at 8-week follow-up.

However, a systematic review by Gaffney et al. [64] identified 13 RCTs of conversational agents for mental health published between 2016–2022. Across studies, small to moderate positive effects were observed for anxiety and depression compared to non-active controls. However, effects compared to active controls (e.g., self-help materials) were considerably smaller and often non-significant. This study highlights the considerable methodological variations across studies, with inconsistent outcome measures, intervention durations, and comparison conditions making direct comparisons challenging. Effect sizes typically diminish when conversational agents are compared to active controls rather than waitlist conditions, suggesting that some benefits may derive from common factors rather than specific conversational AI mechanisms.

Engagement metrics generally exceed those of non-conversational digital interventions, suggesting that the dialogue format may enhance user experience and retention. However, most studies report substantial attrition over time, similar to other digital health interventions. Follow-up periods in most studies remain relatively short (typically 4–8 weeks), with limited evidence regarding maintenance of effects or long-term engagement patterns. This limitation is particularly relevant when considering applications for chronic or recurrent conditions that require sustained support.

Importantly, the therapeutic approaches implemented through conversational AI have been predominantly cognitive behavioral, with fewer examples of other evidence-based approaches such as acceptance and commitment therapy, interpersonal therapy, or mindfulness-based interventions. This focus may reflect both the structured nature of CBT and its established evidence base in digital mental health more broadly.

### 5.2. Emerging Evidence in Pediatric Populations

Research specifically addressing conversational AI applications for children and adolescents has begun to emerge, though it remains less developed than the adult literature. These studies collectively provide important preliminary insights while highlighting significant methodological challenges and knowledge gaps.

Several key themes emerge across the pediatric literature:

- Feasibility and Acceptability Evidence: Multiple studies demonstrate that children and adolescents generally engage well with conversational AI mental health applications. High completion rates (often exceeding 90%) have been reported in short-term feasibility studies [65,66], with qualitative feedback suggesting that young people appreciate the accessibility, privacy, and non-judgmental nature of these interactions. User satisfaction appears particularly strong among adolescents, who value the autonomy and confidentiality these systems provide [67,68].
- Implementation Challenges: Studies exploring real-world implementation have identified several important barriers, including technical difficulties, variable engagement over time, parental concerns about supervision, and integration challenges with existing care systems [68,69]. Dropout rates increase substantially in longer-term implementations, suggesting that initial novelty effects may diminish without ongoing adaptation and support.
- Professional Perspectives: Research examining healthcare providers’ views reveals a consistent pattern of cautious optimism balanced with concerns about clinical oversight and safety. Primary care physicians and mental health specialists generally view conversational AI as potentially valuable for supplementing human care rather than replacing it [8,70]. Professional stakeholders consistently emphasize the need for transparent clinical governance and clear pathways for escalation to human providers when needed.
- Ethical and Developmental Considerations: Several studies specifically examine ethical dimensions of pediatric applications, highlighting tensions between autonomy and protection, privacy and safety, and intended versus actual use patterns [69,71]. These analyses consistently emphasize that developmental considerations must be central rather than peripheral in system design and implementation.
- Limitations of Current Evidence: The pediatric literature shares several methodological limitations with the broader digital mental health field. Sample sizes remain relatively small, with most studies including fewer than 50 participants. Comparison conditions are often absent, making it difficult to distinguish the specific effects of conversational AI from non-specific effects of digital engagement. Follow-up periods are typically brief (2–8 weeks), providing limited insight into sustainable benefits or potential developmental impacts over time.

Furthermore, most studies focus on adolescents rather than younger children, creating a significant knowledge gap regarding applications for elementary and middle school-aged populations. This gap is particularly concerning given the distinct developmental needs and capabilities across childhood and the potential for different interaction patterns with digital systems at various ages.

Table 2 summarizes key studies investigating conversational AI applications in pediatric mental health, highlighting populations studied, methodological approaches, key findings, and limitations. This emerging body of research provides a foundation for more rigorous investigation while emphasizing the need for developmentally informed approaches that address the unique needs of children and adolescents across age ranges.

### 5.3. Therapeutic Mechanisms

Beyond outcome measures, researchers have begun to investigate the mechanisms through which conversational AI may influence mental health. Several potential therapeutic pathways have been identified.

- Reduced Barriers to Self-Disclosure: The perception of anonymity and non-judgment appears to facilitate disclosure of sensitive information that users might hesitate to share with human providers [72]. This fact may be particularly relevant for adolescents navigating identity development and heightened sensitivity to peer evaluation [73].
- Cognitive Change: Structured conversational interventions based on cognitive behavioral principles appear capable of promoting cognitive reframing and challenging maladaptive thought patterns [28]. Text-based interactions may provide opportunities for reflection and cognitive processing that differ from face-to-face exchanges [74].
- Emotional Validation: Analysis of user–chatbot interactions suggests that even simple acknowledgment and reflection of emotions by AI systems can provide a sense of validation that users find supportive [75], aligning with fundamental therapeutic processes identified in human psychotherapy research.
- Behavior Activation: Conversational agents have demonstrated effectiveness in promoting engagement in positive activities and behavioral experiments, core components of evidence-based treatments for depression [76]. The interactive format and ability to send reminders may enhance compliance with behavioral recommendations.
- Skill Development and Practice: Regular interaction with conversational agents provides opportunities for repeated practice of coping skills and emotion regulation strategies in naturalistic contexts [77]. This distributed practice may enhance skill acquisition compared to less frequent traditional therapy sessions.
- Bridging to Human Care: Several studies suggest that conversational agents may serve as “digital gateways” that increase willingness to seek professional help among those who might otherwise avoid traditional services [78]. This bridging function may be especially valuable for adolescents who typically show low rates of help-seeking for mental health concerns [79]. The current evidence suggests that conversational AI applications have promising potential in supporting pediatric mental health, particularly for common conditions like anxiety and depression, and for specific functions such as psychoeducation, skills practice, and bridging to professional care. However, the field remains in its early stages of development, with substantial need for larger, more rigorous studies specifically designed for pediatric populations across developmental stages [80].
- Pediatric-Specific Mechanisms: Several therapeutic mechanisms appear uniquely relevant or modified in pediatric populations. Children and adolescents, as digital natives, often demonstrate greater comfort with technology-mediated communication than adults, potentially facilitating more natural engagement with conversational AI. Research suggests that younger populations may form different types of relationships with non-human entities, with some studies indicating children more readily attribute social presence and therapeutic alliance to AI systems [81]. Age-specific engagement patterns have also been observed, with gamification elements proving particularly effective for younger children, while adolescents often value privacy and autonomy features more highly. Additionally, the reduced power differential between user and AI (compared to adult–child therapeutic relationships) may facilitate different disclosure patterns, particularly among adolescents navigating authority relationships.

## 6. Special Considerations for Pediatric Applications

The application of conversational AI in pediatric mental health contexts requires careful consideration of developmental, ethical, and implementation factors that may differ substantially from adult applications. These special considerations must inform both research and practice to ensure that these technologies appropriately address the unique needs of children and adolescents.

### 6.1. Developmental Considerations

- Cognitive and Language Development: Children’s cognitive and language abilities evolve significantly throughout development, necessitating age-appropriate adjustments to conversational complexity, vocabulary, abstract concepts, and interaction patterns [82]. What works for adolescents may be incomprehensible to younger children, while content designed for younger children may appear patronizing to adolescents.
- Emotional Development: Children’s ability to identify, articulate, and regulate emotions develops gradually, impacting how they express mental health concerns and engage with therapeutic content [83]. Conversational agents must adapt to varying levels of emotional awareness and vocabulary across developmental stages.
- Identity Formation: Adolescence in particular is characterized by intensive identity exploration and formation [84]. Interactions with AI systems during this sensitive period may influence self-concept and beliefs in ways that require careful consideration and safeguards.
- Suggestibility and Critical Thinking: Younger children typically demonstrate greater suggestibility and less developed critical thinking skills, potentially increasing their vulnerability to misinformation or inappropriate advice [85]. This fact necessitates heightened attention to content accuracy and age-appropriate framing of information.
- Digital Literacy: While often characterized as “digital natives”, children and adolescents show significant variation in digital literacy skills that affect their ability to understand AI’s limitations and interpret AI-generated content appropriately [86]. Educational components may be necessary to establish appropriate expectations and boundaries.
- Attention Span and Engagement Preferences: Children’s attention spans and engagement preferences differ from adults and vary across developmental stages, requiring adaptations to conversation length, interaction style, and multimedia integration [87]. Gamification elements may enhance engagement but must be developmentally appropriate [88].

### 6.2. Clinical and Therapeutic Considerations

- Presentation of Mental Health Concerns: Mental health conditions often present differently in children than adults, with more somatic complaints, behavioral manifestations, and developmental impacts [89]. Conversational agents must be trained to recognize and respond appropriately to these pediatric-specific presentations.
- Assessment Challenges: Accurate assessment of mental health in children often requires multi-informant approaches (child, parent, teachers) due to varying perspectives and limited self-awareness [90]. This complicates the design of conversational assessment tools that typically rely on single-user interaction.
- Parental Involvement: Effective mental health interventions for children generally involve parents/caregivers, raising questions about how conversational AI should manage family involvement while respecting the child’s growing autonomy and privacy needs [91]. Different models of parent–child–AI interaction may be needed across developmental stages.
- Comorbidity and Complexity: Children with mental health concerns frequently present with comorbid conditions or complex contextual factors that may exceed the capabilities of narrowly focused conversational interventions [92]. Clear pathways for escalation to human providers are essential when complexity emerges.
- School Context: For many children, mental health supports are accessed primarily through educational settings rather than healthcare systems [93]. This suggests the potential value of developing conversational agents specifically designed for school-based implementation with appropriate integration into existing support structures.
- Illness Severity Considerations: Conversational AI applications must incorporate robust assessment of symptom severity with clear protocols for cases requiring higher levels of care. Certain conditions such as active suicidality, psychosis, severe eating disorders, or substance use disorders typically require immediate human intervention and may be inappropriate for stand-alone AI management. Systems must be designed to recognize their limitations, effectively triage based on severity, and facilitate appropriate referrals when needed. This consideration is particularly important in pediatric contexts where symptom presentation may differ from adults and where certain high-risk behaviors require mandatory reporting obligations [94].

### 6.3. Safety and Ethical Considerations

- Content Safety: Heightened responsibility exists for ensuring age-appropriate content and preventing exposure to harmful, frightening, or developmentally inappropriate information [95]. Careful consideration of how mental health concepts are explained and discussed must be considered.
- Crisis Detection and Response: Robust protocols for detecting and responding to crisis situations, including suicidality, abuse disclosure, or emergent safety concerns, are particularly critical in pediatric applications [96]. Clear pathways for human intervention must exist when necessary.
- Privacy and Confidentiality: Complex balancing is required between respecting the growing need for privacy among older children and adolescents and ensuring appropriate adult oversight for safety and care coordination [97]. Different approaches may be needed across age groups and risk levels.
- Data Protection: Special protections apply to children’s data under various regulatory frameworks (e.g., COPPA in the US, GDPR in Europe), necessitating stringent data handling practices and transparent communication about data usage [98]. Beyond regulations specifically addressing children’s data, health information shared with conversational AI should be considered protected health information under regulations such as HIPAA in the US, requiring appropriate security measures, breach notification protocols, and limitations on data use and disclosure.
- Developmental Impact: Long-term effects of regular interaction with AI systems during critical developmental periods remain largely unknown, necessitating ongoing monitoring and research to identify potential unintended consequences [99].
- Autonomy and Agency: The complex patchwork of laws regarding minor consent to mental health treatment creates significant challenges for AI deployment across different regions. Age thresholds vary widely—from 13 in Washington state to 16 in Texas and 18 in many other jurisdictions—requiring systems to implement location-specific protocols for consent, parental involvement, and information sharing. Respect for developing autonomy requires giving children appropriate voice in decisions about using AI mental health tools while acknowledging their evolving capacity for informed consent [100]. This balance shifts across developmental stages.

### 6.4. Implementation Considerations

- Access and Equity: Digital divides affect children disproportionately, with socioeconomic factors influencing access to devices, internet connectivity, and private spaces for sensitive conversations [101]. Implementation strategies must address these disparities to avoid exacerbating existing inequities.
- Integration with Support Systems: For maximal effectiveness, conversational AI applications for children should integrate with existing support ecosystems, including schools, primary care, mental health services, and family systems [102]. Standalone applications may have limited impact without these connections.
- Cultural Responsiveness: Children develop within specific cultural contexts that shape understanding of mental health, help-seeking behaviors, and communication styles [103]. Conversational agents must demonstrate cultural humility and adaptability to diverse perspectives.
- Supervised vs. Independent Use: Decisions about whether and when children should engage with mental health AI independently versus under adult supervision require balancing safety concerns with developmental needs for privacy and autonomy [104]. Graduated independence may be appropriate across age ranges.
- Educational Support: Implementation in pediatric contexts may require more substantial educational components for both children and adults to establish appropriate expectations, boundaries, and understanding of AI limitations [105].

These special considerations highlight the complexity of developing and implementing conversational AI for pediatric mental health. Rather than simply adapting adult-oriented systems, developmentally appropriate applications require fundamental reconsideration of design principles, content, interaction patterns, safety protocols, and implementation strategies [106]. Successfully addressing these considerations requires interdisciplinary collaboration among developmental psychologists, pediatric mental health specialists, ethicists, technology developers, and—critically—children and families themselves.

## 7. Discussion

This narrative review aims to map the emerging landscape of conversational AI applications in pediatric mental health, highlighting both promising potential and significant challenges that must be addressed for responsible implementation. Several key themes have emerged across the literature that warrant further discussion.

### 7.1. The Promise and Limitations of Current Evidence

The evidence base for conversational AI in pediatric mental health remains nascent, with most robust empirical studies focused on adult populations. While preliminary research suggests potential benefits for addressing common mental health conditions like anxiety and depression [107], significant gaps exist in the pediatric literature. The studies that do exist with children and adolescents are predominantly small-scale feasibility or acceptability studies rather than rigorous effectiveness trials. This pattern mirrors the historical development of digital mental health interventions more broadly, where adult applications typically precede pediatric adaptations.

The therapeutic mechanisms identified in the literature—including reduced barriers to self-disclosure, cognitive change, emotional validation, behavioral activation, skill development, and bridging to human care—align with established principles of effective mental health interventions [108]. However, the degree to which these mechanisms operate similarly across developmental stages remains largely theoretical rather than empirically established. The field must move beyond extrapolating from adult findings to directly investigating how these mechanisms function within specific developmental contexts.

### 7.2. Developmental Appropriateness as a Fundamental Challenge

Perhaps the most consistent theme across the literature is the critical importance of developmental appropriateness in conversational AI applications for children and adolescents. Unlike many digital adaptations that require primarily interface modifications for younger users, conversational AI requires fundamental reconsideration of multiple dimensions, including language complexity, cognitive processing, emotional development, and interaction patterns [109]. The significant variation in capabilities across childhood and adolescence further complicates this challenge, suggesting that a developmental spectrum of conversational AI applications may be necessary rather than a single pediatric approach.

Current conversational AI systems, particularly those based on large language models, are trained predominantly on adult-oriented text corpora that may poorly represent children’s communication patterns, concerns, and developmental needs [110]. This foundational limitation highlights the need for specialized training approaches and careful adaptation of existing systems for pediatric applications.

### 7.3. Balancing Innovation with Safety and Ethics

The literature reveals the tension between the imperative for innovation to address significant unmet needs in pediatric mental health and the heightened ethical responsibilities when developing technologies for vulnerable young populations [111]. While conversational AI offers potential solutions to persistent barriers in traditional care models—including accessibility, stigma, and resource constraints—these benefits must be weighed against risks, including privacy concerns, potential for harmful content, and the largely unknown developmental impacts of regular AI interaction.

Existing frameworks for digital ethics in healthcare typically address adult populations and may inadequately account for children’s unique vulnerabilities and evolving capacities. The development of pediatric-specific ethical guidelines for conversational AI represents an urgent need, with particular attention to issues of informed consent, privacy boundaries between children and parents/guardians, crisis detection protocols, and safeguards against potential developmental harm [112].

The rapidly evolving legal landscape surrounding AI, mental health data, and minor consent creates significant implementation challenges. Developers must navigate not only healthcare regulations but also emerging AI-specific legislation that may impose additional requirements for transparency, explainability, and human oversight, particularly for applications involving vulnerable populations like children. These legal considerations directly impact the design, deployment, and governance of conversational AI in pediatric mental health contexts, necessitating interdisciplinary collaboration between legal experts, ethicists, clinicians, and developers.

### 7.4. Integration Rather than Replacement

The literature consistently emphasizes that conversational AI should function as a complement to, rather than replacement for, human-delivered mental health support [113]. The most promising implementations position AI within comprehensive ecosystems of care rather than as standalone interventions, integrating with schools, primary care, specialized mental health services, and family systems [80]. This integration approach acknowledges both the capabilities and limitations of current AI technologies while leveraging existing support structures to maximize benefits and mitigate risks.

For children in particular, the relational context of mental health support appears fundamentally important, suggesting that hybrid models combining AI and human elements may prove most effective [114]. These might include AI systems that facilitate connection to human providers, augment existing therapeutic relationships, or operate under various levels of human supervision depending on the child’s age, needs, and risk level.

### 7.5. Equity and Access Considerations

While conversational AI may potentially address some access barriers in traditional mental healthcare, the literature highlights concerns that without deliberate attention to equity, these technologies could exacerbate existing disparities [115]. Digital divides affecting device access, internet connectivity, and digital literacy disproportionately impact children from socioeconomically disadvantaged backgrounds, potentially limiting the reach of AI-based interventions to those with greater resources.

Additionally, current conversational AI systems demonstrate limitations in cultural responsiveness and linguistic diversity that may particularly affect children from minoritized communities [116]. These limitations include reduced performance in non-dominant languages, cultural biases in content and interaction styles, and inadequate representation of diverse cultural conceptualizations of mental health and wellbeing. Addressing these equity concerns requires intentional design approaches that prioritize accessibility, cultural humility, and linguistic inclusivity from inception rather than as afterthoughts. Implementation strategies that specifically target underserved populations and contexts where traditional mental health resources are most limited are necessary.

### 7.6. Research Gaps and Future Directions

This narrative review has identified several critical gaps in the current literature that should guide future research.

Developmental Validation: Studies explicitly examining how children at different developmental stages interact with and respond to conversational AI, including potential differences in engagement patterns, comprehension, trust formation, and therapeutic benefit.

Longitudinal Outcomes: Research investigating the medium- to long-term impacts of conversational AI interventions on pediatric mental health outcomes, including potential maintenance effects, habituation, and developmental influences.

Implementation Science: Studies examining how conversational AI can be effectively integrated into existing systems of care, including schools, primary care, and specialized mental health services.

Safety Monitoring: Systematic approaches to identifying and mitigating potential harms associated with conversational AI in pediatric populations, including protocols for crisis detection, content safety, and developmental impact assessment.

Equity-Focused Design and Evaluation: Research explicitly addressing how conversational AI can be designed and implemented to reduce rather than exacerbate health disparities across socioeconomic, cultural, and linguistic dimensions.

Comparative Effectiveness: Studies directly comparing conversational AI to established interventions and examining potential differential effects across developmental stages, presenting problems, and implementation contexts.

Addressing these research priorities will require interdisciplinary collaboration among developmental psychologists, pediatric mental health specialists, computer scientists, implementation researchers, ethicists, and—critically—children and families themselves. Participatory research approaches that meaningfully involve young people in design and evaluation processes may be particularly valuable for ensuring that conversational AI applications meet their needs and preferences [117].

## 8. Conclusions

This narrative review addressed five key research questions regarding conversational AI in pediatric mental health:

1. What types of conversational AI applications have been developed for pediatric mental health? Our review identified diverse applications spanning rule-based, retrieval-based, generative, and hybrid systems. These technologies support functions including screening and assessment, psychoeducation, guided self-help, emotional support, and augmentation of traditional therapy. The most promising implementations are those designed specifically for pediatric populations rather than adapted from adult systems, with developmental considerations integrated throughout the design process.
2. What is the current evidence regarding effectiveness, acceptability, and safety? The evidence base remains nascent, with most robust empirical studies focused on adult populations. Preliminary research with pediatric populations shows promising engagement metrics and user satisfaction, particularly for common conditions like anxiety and depression. However, efficacy studies with rigorous methodology, appropriate controls, and sufficient sample sizes are largely absent. Safety protocols appear inconsistent across implementations, with limited systematic evaluation of potential harms or unintended consequences.
3. What unique considerations apply to children and adolescents compared to adults? Developmental considerations emerged as fundamental rather than peripheral factors, necessitating adaptations across cognitive, linguistic, emotional, and ethical dimensions. Children’s evolving capabilities for abstract thinking, emotional regulation, and decision-making require age-appropriate content and interaction patterns. The need for parental involvement balanced with growing autonomy presents unique challenges, as do the complex legal frameworks governing minor consent and data protection across jurisdictions.
4. What ethical, technical, and implementation challenges have been identified? Critical challenges include ensuring privacy and confidentiality while enabling appropriate oversight, developing robust crisis detection and response protocols, addressing digital divides that may limit access, ensuring cultural responsiveness, and integrating systems with existing support networks rather than creating standalone interventions. Technical challenges involve adapting language models predominantly trained on adult text to pediatric communication patterns and ensuring age-appropriate content filtering.
5. What gaps exist in the current research landscape? Substantial gaps include the need for developmental validation studies examining how children at different stages interact with conversational AI, longitudinal outcomes research assessing medium- to long-term impacts, implementation science approaches for integration into care systems, rigorous safety monitoring protocols, and equity-focused design and evaluation to ensure these technologies reduce rather than exacerbate existing disparities.

Conversational AI shows potential as a valuable component in addressing significant unmet mental health needs among children and adolescents. However, its appropriate role appears to be as a complement to, rather than replacement for, human-delivered care. With thoughtful development centered on children’s unique needs, interdisciplinary collaboration, and continued empirical evaluation, these technologies may help create more accessible, engaging, and effective mental health support systems for young people worldwide.

## Figures and Tables

**Table 2:** Selected studies investigating conversational AI applications in pediatric mental health, highlighting populations studied, methodological approaches, key findings, and limitations.

Study	Population	AI System & Therapeutic Approach	Study Design	Key Findings	Limitations
Vertsberger et al. (2023) [65]	Adolescents	Kai.ai (Acceptance Commitment Therapy)	Longitudinal study	Improvements in stress management and emotional well-being	Self-reporting biases; Lack of long-term follow-up
Papneja et al. (2024) [72]	Youth (various ages)	Multiple systems, including Wysa (CBT)	Systematic review	AI valuable as supplement to traditional therapy; Cannot replace human clinicians	Limited long-term efficacy data
Nicol et al. (2023) [67]	Adolescents with depression and anxiety	CBT-based chatbot	Feasibility and acceptability study	High engagement; Perceived helpfulness	Small sample size; No control group
Ghadiri et al. (2024) [70]	Primary care physicians providing adolescent mental healthcare	N/A (Physicians’ perceptions of AI)	Qualitative study	Skepticism about AI reliability; Recognition of potential as supplementary tool	Limited to physician perspectives
Fujita et al. (2023) [68]	Adolescents on psychiatric waiting lists	Emol	Implementation study	Identified technical challenges and dropout rates	Focus on implementation rather than outcomes
Beaudry et al. (2019) [66]	Teenagers	Mood management conversational agent	Feasibility study	97% completion rate; High user satisfaction	Small sample; No efficacy measures
Moore et al. (2025) [71]	Children and adolescents	Various AI chatbots	Ethical analysis	AI should support rather than replace therapy; Concerns about parental consent	Limited empirical data
Opel et al. (2024) [69]	Adolescents	AI mental health therapist systems	Systematic analysis	Improved accessibility; Need for regulation to mitigate risks	Theoretical focus; Limited experimental evidence

## Data Availability

Data available upon reasonable request to corresponding author.

## Abbreviations

The following abbreviations are used in this manuscript:

AI	Artificial Intelligence
LLM	Large Language Model
CBT	Cognitive Behavorial Therapy
ACT	Acceptance Commitment Therapy
NLP	Natural Language Processing
RCT	Randomized Controlled Trial
COPPA	Children’s Online Privacy Protection Act
GDPR	General Data Protection Regulation
ELIZA	Early Natural Language Processing Computer Program
